# Results of stapes surgery for otosclerosis with two kinds of prothesis in residency training

**DOI:** 10.1016/S1808-8694(15)30142-7

**Published:** 2015-10-18

**Authors:** Celso Dall”Igna, Vanessa Niemiec Teixeira, Daniela Pernigotti Dall”Igna, Letícia Petersen Schmid Rosito

**Affiliations:** a PhD in Medicine at UFRGS, Adjunct Professor of Otorhinolaryngology at UFRGS, Head of the ENT service at Hospital de Clínicas de Porto Alegre; b Otorhinolaryngologist, MSc student at UFRGS, Fellow in Otology at Hospital de Clinicas de Porto Alegre; c Resident Physician in Otorhinolaryngology at Hospital das Clínicas da Universidade Federal do Paraná; d MSc in Otorhinolaryngology at UFRGS, Physician at Hospital de Clínicas de Porto Alegre; e Hospital de Clínicas de Porto Alegre, Universidade Federal do Rio Grande do Sul

**Keywords:** deafness, otosclerosis, stapedectomy, conductive hearing loss

## Abstract

Stapes surgery is one of the approaches indicated to treat conductive hearing loss secondary to otosclerosis. The procedures requires skill and experience from the surgeon and is part of medical residency training. **Aims**: To assess which type of prosthesis (Teflon or metal/steel) presents the best results in surgeries performed by residents and the incidence of complications. **Materials and methods**: we retrospectively assessed 189 interventions that counted on the active participation of resident physicians, and we compared the two types of prosthesis used. Audiometric results were analyzed following the guidelines from the Committee on Hearing and Equilibrium and also according to the Amsterdam Hearing Evaluation Plots. **Results**: Bone-air gap reduced in an average value of 21.90 dB (p<0.05) after the surgery in the group that received the Teflon prosthesis and 21.37 dB (p<0.05) in the group that received the mixed prosthesis, and gain in SRI was of 22.33 and 26.10 dB (p<0.05), and the air-bone gap was below 20 dB in 80.6% and 85.04%, respectively. **Conclusions**: We did not see differences in the audiometry and in the incidence of complications when we compared the type of prosthesis used. We believe it is valid to continue teaching this procedure in medical residency training programs, regardless of the type of prosthesis.

## INTRODUCTION

Otospongiosis is a disease characterized by otic involvement due to primary bone metabolism dystrophy. This condition often promotes the fixation of the stapes on the oval window, resulting in conductive sensorial or mixed hypoacusis. It is a dominant autosomal hereditary disease with incomplete penetrance (approximately 40%) and varied expression. Stapediovestibular anchylosis was described for the first time in an autopsy conducted by Antonio Valsalva in 1753. Over a century later Kessekl performed the first stapes mobilization procedure. Only in 1958 did John Shea introduce surgery as an option to treat otospongiosis using prosthesis, in a popular technique that has been modified up until current days^1^, ^2^, ^3^, ^4^, ^5^, ^6^, ^7^, ^8^, ^9^.

Since it was described for the first time, a number of materials have been used in the making of the prosthetic devices, but no significant differences have been found in the audiological results provided by them. Research on the subject stems from surgical procedures conducted by experienced hands^10^, ^11^, ^12^, ^13^.

Reports on this procedure performed by resident physicians have consistently indicated inferior results when compared to the outcomes produced by experienced surgeons. Some authors consider it safe to offer this procedure in medical residence services, while others dispute that conclusion^14^, ^15^, ^16^, ^17^.

The objective of this study is to check whether there is a difference in surgical outcome between the use of two commonly employed prosthetic implants in otospongiosis surgery (Teflon - group I and mixed implant metal + Teflon - group II). Patient pre and postoperative audiometric thresholds were analyzed, as well as surgery complications. All procedures were performed by resident physicians trained at a university hospital.

## MATERIALS AND METHOD

This is a retrospective study that looked into the records of patients submitted to otospongiosis surgery between May of 1988 and August of 2005. Only the records containing a complete description of the procedure, pre and postoperative audiometric data, and at least one year of follow-up were selected. Ears previously operated on were excluded. All 189 procedures were done under general anesthesia and orotracheal intubation in hospitalized patients as part of the training of medical residents of the university hospital. Residents were directly supervised either by the author (CD) of one of the three surgeon professors at the service.

The data sets obtained from patient chart review were compiled in a protocol. Identification information (birth date, gender, ethnicity), data pertaining to the surgical procedures (operated ear, type of procedure, previous surgery on the same ear), intra and postoperative complications, tone audiometry test results (for 0.25, 0.5, 1, 2, 3, 4, 6 and 8kHz for air conduction and 0.5, 1, 2, 3 and 4kHz for bone conduction) and speech audiometry tests done before and after surgery were gathered. An Amplaid AD27 audiometer was used in audiometric tests along with a soundproof booth and speech therapists hired by our service. When bone or air conduction thresholds could not be measured in a certain frequency it was registered as non-measurable; a value of 10dB above the device”s capacity was considered the threshold^18^.

Each surgical procedure was analyzed separately. Some patients were included more than once in the study, as they were operated in both ears (n=51). Teflon implants were used in 67 procedures, while the mixed device was used in 122 cases.

Patients were aged between 16 and 77 years at the time of surgery (mean 42±10); 69.8% were females and 97.3% Caucasian. Mean postoperative follow-up was 22.8 (±26.57) months.

Pre and postoperative audiometry test results were compared in the following terms: (1) threshold for each frequency, separately for air and bone conduction; (2) threshold average for 0.5, 1, 2 and 3kHz for air and bone conduction was produced as defined by the Committee on Hearing and Equilibrium^19^ to present the results related to conductive hearing loss; the average between the thresholds at 2 and 4kHz was used for cases where thresholds for 3kHz were not available18; (3) air-bone gap obtained by the difference between air and bone conduction threshold averages; and (4) speech recognition index (SRI). Patient records without pre or postoperative data on tone or speech audiometry were excluded.

The results, as well as surgery success rates, are presented in the form of the Amsterdam Hearing Evaluation Plots (AHEPs) proposed by De Bruijn et al.^20^. Surgical outcomes were considered excellent when postoperative air conduction was better than preoperative bone conduction (improved bone thresholds); satisfactory when the difference between postoperative air conduction and preoperative bone conduction was lower than 20dB; all other outcomes were deemed unsatisfactory.

Data statistical analysis was performed using Student”s t-test for paired samples; statistically significant differences were elicited when p≤0.05. Data sets were compiled and analyzed with the aid of software program SPSS for Windows 10.0.

This study was approved by the Ethics Committee of our university hospital under permit 078/05.

## RESULTS

**Figure 1:**
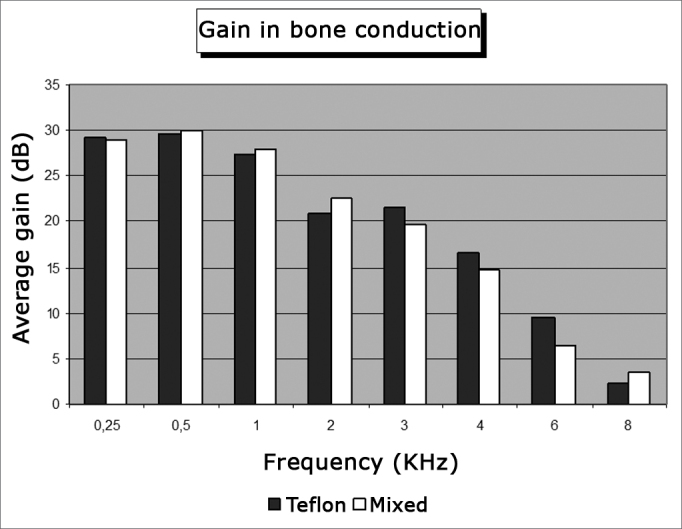
Average air conduction gain by frequency.

**Figure 2:**
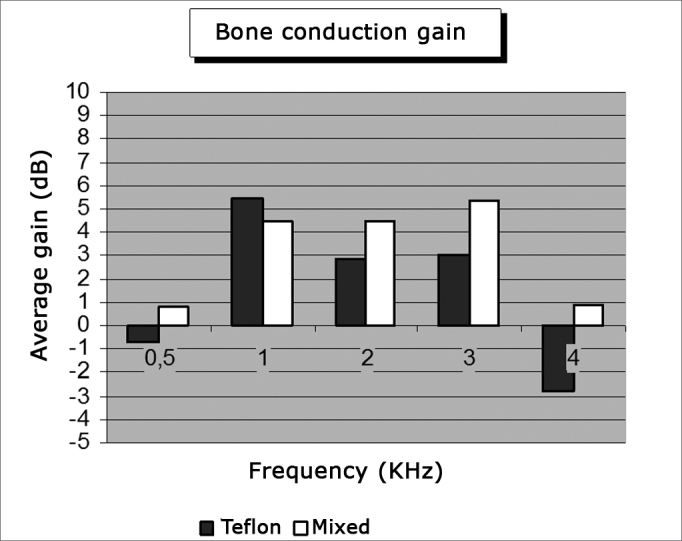
Average bone conduction gain by frequency.

**Figure 3:**
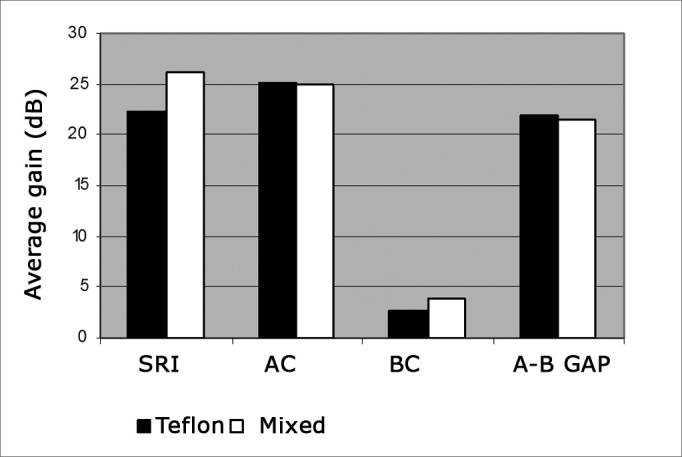
Average gains in air conduction (CA); bone conduction (CO); air-bone gap (GAP A-O) and speech recognition index (IRF).

Table 1 shows the mean values for pre and postoperative air and bone conduction thresholds, air-bone gap, and SRI (speech recognition index). The first data set belongs to patients using Teflon implants (group I), while Table 2 presents the data for patients using mixed Teflon and metal implants (group II). There was statistically significant threshold improvement for all frequencies in air conduction, except 8kHz (Fig. 1). Significant improvement was also observed in bone conduction for 1, 2, and 3kHz, but in none of them the improvement exceeded 4 dB (Fig. 2). On average, the overall gain in air conduction was of 25.18 dB (±22.53 p<0.05) for group I and of 24.99 dB (±15.3 p<0.05) for group II. Average bone conduction gain amounted to 2.56dB (p<0.05) for group I and 3.94 dB (p<0.05) for group II. Air-bone gap was reduced on average by 21.90 dB (±14.85 p<0.05) after surgery in group I and by 21.37 dB (±12.12 p<0.05) in group II. Speech audiometry test results were available for 151 ears of the 189 procedures, 60 of which in group I and 91 in group II. Average SRI gain was of 22.33 dB (±22.45 p<0.05) in group I and 26.10 (±14.79 p<0.05) in group II (Fig. 3). No statistically significant difference was found in comparing the results from both groups (p>0.05).

**Table 1:** Pre and postoperative thresholds of air and bone conduction, air-bone gap, and SRI. Group I - Teflon.

Frequency (kHz)	N	Preoperative	Postoperative	Difference	P†
Air conduction					
0,25	64	65,55 ± 13,25	36,25 ± 23,13	29,30 ± 21,21	<0,001
0,5	64	65,23 ± 12,03	35,55± 24,43	29,69 ± 22,14	<0,001
1	64	62,97 ± 12,59	35,63 ± 23,44	27,34 ± 22,73	<0,001
2	64	57,97 ± 14,13	36,95 ± 23,10	21,02 ± 23,64	<0,001
3	62	58,39 ± 15,93	36,94 ± 24,40	21,45 ± 25,90	<0,001
4	64	60,55 ± 19,25	44,06 ± 24,57	16,48 ± 26,75	<0,001
6	64	61,72 ± 18,54	52,27 ± 26,53	9,45 ± 26,93	0,03
8	63	60,24 ± 20,78	57,94 ± 27,79	2,30 ± 27,00	0,157
Mean values^1^	62	61,07 ± 11,93	35,89 ± 22,59	25,18 ± 22,53	<0,001
Bone conduction					
0,5	63	21,11 ± 9,65	21,83 ± 14,90	-0,71 ± 14,48	0,532
1	64	28,36 ± 9,04	22,89 ± 14,96	5,47 ± 14,13	<0,001
2	64	31,80 ± 13,64	28,91 ± 16,94	2,89 ± 14,66	0,011
3	63	31,90 ± 13,12	28,89 ± 17,86	3,02 ± 17,24	0,023
4	64	28,59 ± 14,76	31,41 ± 19,67	-2,81 ± 18,30	0,685
Mean values^1^	62	32,08 ± 13,22	29,95 ± 15,01	2,56 ± 13,57	<0,001
Air-bone gap^2^	60	32,77 ± 8,05	10,88 ± 12,47	21,90 ± 14,85	<0,001
SRI Vocal audiometry^3^	60	62,67 ± 13,16	40,33 ± 21,13	22,33 ± 22,45	<0,001

The data represent mean ± standard deviation, in decibels.

† Significance level (Student t test for paired samples) of air and bone conduction threshold differences.

1 Mean values among the frequencies of 0.5, 1, 2 and 3 kHz. When not available, the 3kHz threshold was replaced by the arythmetical average of the 2 and 4 kHz frequencies.

2 Difference between the air and bone conduction mean values.

3 Speech Recognition Index.

**Table 2:** Pre and postoperative thresholds of air and bone conduction, air-bone gap, and SRI. Group II - Mixed.

Frequency (kHz)	N	Preoperative	Postoperative	Difference	P†
Air conduction					
0,25	107	66,96 ± 13,56	37,99 ± 19,25	28,97 ± 18,22	<0,001
0,5	107	65,89 ± 14,60	35,84 ± 20,37	30,04 ± 16,94	<0,001
1	107	64,49 ± 13,73	36,59 ± 21,69	27,90 ± 17,33	<0,001
2	107	60,14 ± 15,44	37,48 ± 22,04	22,66 ± 16,02	<0,001
3	106	58,21 ± 17,27	38,63 ± 24,68	19,58 ± 17,14	<0,001
4	107	58,74 ± 18,44	44,07 ± 23,57	14,67 ± 17,24	<0,001
6	104	62,07 ± 20,55	55,63 ± 28,26	6,44 ± 19,05	<0,001
8	107	60,93 ± 22,78	57,38 ± 27,27	3,55 ± 15,77	0,010
Mean values^1^	106	62,17 ± 13,07	37,18 ± 20,95	24,99 ± 15,30	<0,001
Bone conduction					
0,5	107	20,14 ± 11,29	19,35 ± 12,21	0,79 ± 8,50	0,177
1	107	29,91 ± 12,70	25,47 ± 16,49	4,44 ± 10,80	<0,001
2	107	33,18 ± 15,61	28,74 ± 17,38	4,44± 10,38	<0,001
3	101	33,71 ± 15,98	28,32 ± 18,82	5,40 ± 10,31	<0,001
4	107	29,72 ± 17,40	29,07 ± 18,77	0,85 ± 9,98	0,306
Mean values^1^	101	29,21 ± 12,29	25,26 ± 14,41	3,95 ± 7,36	<0,001
Air-bone gap^2^	100	32,90 ± 7,80	11,53 ± 10,35	21,37 ± 12,12	<0,001
SRI Vocal audiometry^3^	91	65,33 ± 10,48	39,23 ± 16,88	26,10 ± 14,79	<0,001

The data represent mean value ± standard deviation, in decibels.

† Level of significance (Student t test for paired samples) of the air and bone conduction difference thresholds.

1 Mean values among the frequencies of 0.5, 1, 2 and 3 kHz. When not available, the 3kHz threshold was replaced by the arythmetic average of frequencies 2 and 4 kHz.

2 Air and bone conduction average values difference.

3 Speech Recognition Index.

**Figure 4:**
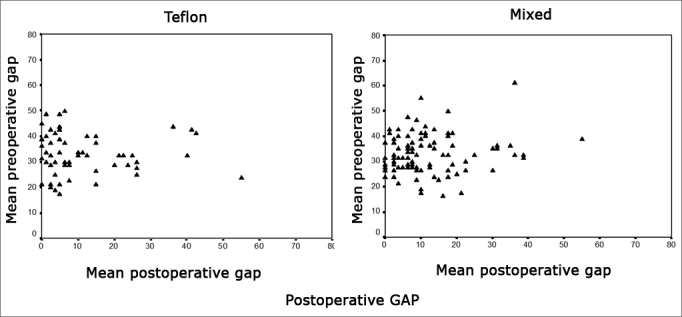
Postoperative gap.

In preoperative care, 93.85% and 94.74% of the ears on groups I and II respectively had air-bone gaps exceeding 20 dB. After surgery the gap was reduced by 20dB or less in 80.6% and 85.04% of the ears, and by 10dB or less in 66.13% and 60.7% of the ears belonging to groups I and II respectively. Only 19.3% and 14.9% of patients in groups I and II respectively had gap values greater than 20 dB (Fig. 4). There was no significant difference between the values observed in both groups (p>0.05).

In all frequencies improvements were observed in air conduction in relation to preoperative conditions; for 8kHz such difference was not statistically significant (p>0.05). Bone conduction was also improved; differences were not statistically significant for 0.5kHz and 4 kHz. Air-bone gap for all frequencies and SRI improved after surgery (p<0.05), and no significant differences were seen between groups (p>0.05).

**Figure 5:**
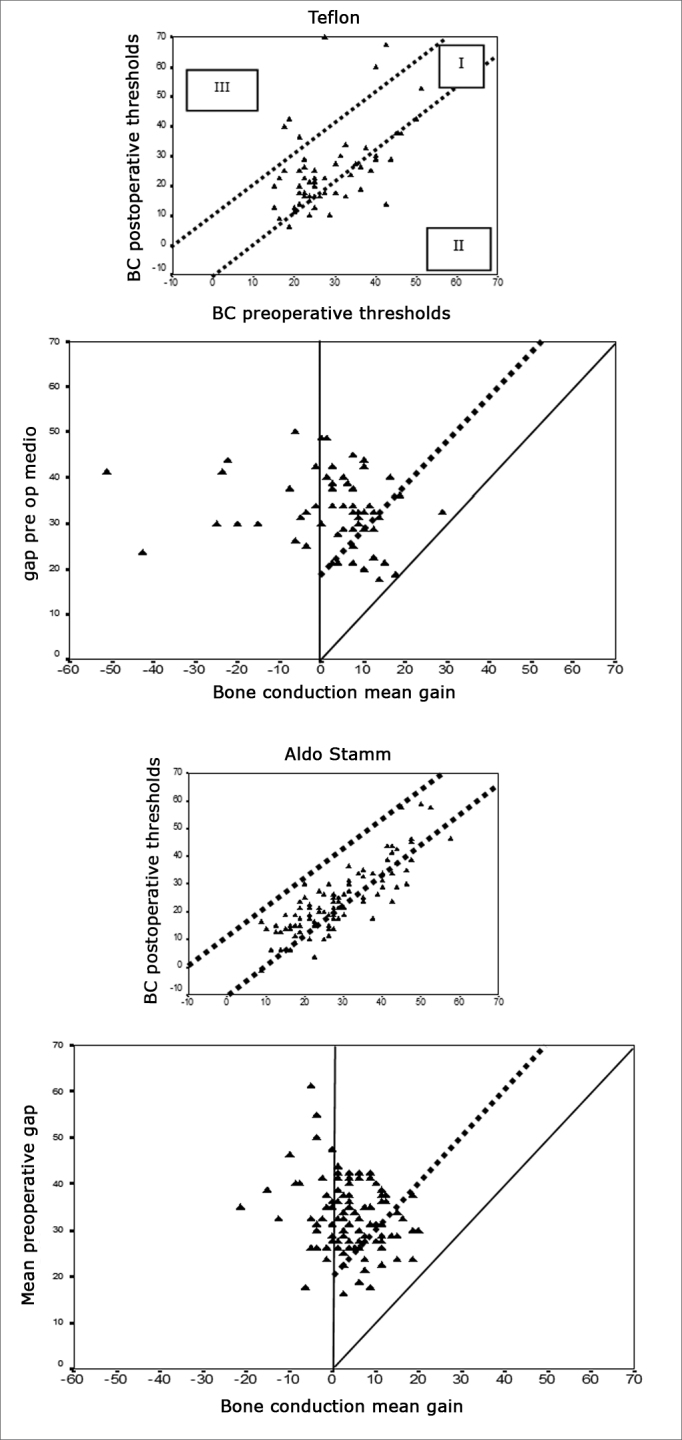
Audiometric outcome of surgical procedures viewed with AHEPS. First for Teflon implant and second for mixed implant. A - pre and postoperative Bone Conduction (VO) tracing per operated ear. The two diagonal lines contain the areas with bone conduction not varying more than 10dB (area I). II - bone conduction improvement due to Cahart effect; III - cochlear damage (decrease in bone conduction > 10dB). B. Postoperative bone conduction gain against preoperative air-bone gap for individual ears. The solid diagonal line indicates total closure of the difference between preoperative air and bone conduction values. Surgical failure - negative alteration in air conduction or air conduction alteration not significant enough to close the difference between pre and postoperative air conduction to less than 20 dB - values above dotted line. Under the solid line are the values in which air conduction gain was greater than expected from the preoperative gap (”overclosure”).

Considering the success criteria defined by the AHEPs, failure occurred in 7.5% and 17.2% of the cases in groups I and II respectively; difference between groups was not statistically significant (p>0.05) (Fig. 5).

The following postoperative complications were observed: 5 cases of tympanic membrane perforation (one in group I and four in group II), 2 cases of permanent vertigo, both in group II, 7 cases of deep hypoacusis, 3 (4%) in group I and 4 (3.2%) in group II.

## DISCUSSION

Stapedotomy is the procedure of choice when treating patients with conductive hearing loss secondary to stapedial otospongiosis. In our university hospital, resident surgeons participate in the procedure in their third year of training under the direct supervision of a professor. Before participating more actively, they follow procedures performed by experienced surgeons and train for the procedure in the temporal bone dissection lab. All procedures are done under general anesthesia so that training surgeons are more at ease and to reduce concerns associated with surgery length.

Improvements were observed in air conduction after surgery in both groups, although in the lower limit this finding agrees with the literature, in which improvements between 22dB and 32dB in air conduction are reported. All analyzed frequencies but 8kHz presented statistically significant differences between pre and postoperative thresholds. This pattern of important improvement in the lower frequencies, less accentuated at 4kHz, and little or no improvement in higher frequencies (6kHz and 8kHz), is in agreement with the literature^10^, ^11^, ^12^, ^13^, ^14^, ^15^, ^16^, ^17^, ^18^, ^21^, ^22^, ^23^, ^24^, ^25^.

In bone conduction the difference between the pre and postoperative threshold averages for 1kHz, 2kHz and 3kHz was statistically significant. This is probably due to the large size of the sample and not to clinical differences, as is observed for example in the 2kHz frequency, in which average improvement was only of 3dB to 4dB depending on the group. Similar findings were reported by other authors^10^, ^11^, ^12^, ^13^, ^14^, ^15^, ^16^, ^17^, ^18^, ^20^, ^21^, ^24^. Cochlear damage was found in 3.2% and 4% of the cases in groups I and II respectively, more than De Bruijn et al.^18^ reported and similar to the levels described ported by Frías et al.^21^.

Otospongiosis surgery presents better results in air conduction on lower frequencies (0.5kHz to 3kHz). In some patients it may even enhance sensorineural hearing perception.

There is wide variation in the audiometric criteria used to define surgical success and consequently many difficulties in comparing results between different authors. In our study we tried to minimize disparity by using the guidelines issued by the Committee on Hearing and Equilibrium^18^ and the method proposed by De Bruijn et al.^19^. Both seemed quite adequate to allow for between-study comparison. This method allows the effect of surgery in each ear to be deduced individually. It also allows favorable and unfavorable results to be easily identified.

Looking at surgery success from the standpoint of air-bone gap, as proposed by a number of authors^21^, ^24^, ^25^, we were able to have air-bone gaps equal to or lower than 10dB in 66.13% and 60.7% of the cases, and lower or equal to 20dB in 80.6% and 85.04% of the ears, for groups I and II respectively. It is difficult to compare our findings to those in the literature given the multitude of ways in which results are presented.

Considering the success criteria defined by the AHEPs, our failure rates amounted to 7.5% and 17.2% in groups I and II respectively; these values are similar to what was reported by De Bruijn et al. (10.9%) in relation to our group I and nearly half of what we found for group II17. This can be explained by the lack of experience of the surgeons on charge of the procedure.

Given the complications it introduced in communication, speech audiometry (SRI) should be more appreciated in the success analysis of ear surgery. In the cases revised in this study, SRI gains were greater than 20 dB in 70% of group I and in 68.13% in group II subjects. Gains exceeding 10dB were seen in 73.3% and 83.5% of groups I and II respectively.

No difference was found when comparing audiological results of patients using Teflon or mixed implants, as also reported in the literature for surgery done by experienced surgeons. No difference was found for complication rates either. However, as this is a retrospective study based on patient chart data, complication rates tend to be underestimated. This could also explain the disagreement between studies in terms of intra and postoperative complications.

## CONCLUSION

As seen in the literature, no differences were observed on the type of implant used in stapedial surgery, although audiological results were worse than those described in the literature for experienced surgeons. Complication rates were not higher than values published in the literature. We believe this procedure should be offered as part of the training of resident surgeons, as long as they receive previous training on temporal bone dissection, follow experienced surgeons during surgery, and are continuously supervised by an advisor throughout the procedure.
